# Comprehensive Review of Knee Osteoarthritis Pharmacological Treatment and the Latest Professional Societies’ Guidelines

**DOI:** 10.3390/ph14030205

**Published:** 2021-03-02

**Authors:** Dragan Primorac, Vilim Molnar, Vid Matišić, Damir Hudetz, Željko Jeleč, Eduard Rod, Fabijan Čukelj, Dinko Vidović, Trpimir Vrdoljak, Borut Dobričić, Darko Antičević, Martina Smolić, Mladen Miškulin, Damir Ćaćić, Igor Borić

**Affiliations:** 1 St. Catherine Specialty Hospital, 49210 Zabok/10000 Zagreb, Croatia; vilim.molnar@svkatarina.hr (V.M.); vid.matisic@svkatarina.hr (V.M.); ortohud@gmail.com (D.H.); zeljko.jelec@svkatarina.hr (Ž.J.); eduard.rod@svkatarina.hr (E.R.); fabijan.cukelj@svkatarina.hr (F.Č.); dinko.vidovic@gmail.com (D.V.); trpimir.vrdoljak@svkatarina.hr (T.V.); dobricic_borut@yahoo.de (B.D.); darko.anticevic@gmail.com (D.A.); igor.boric@svkatarina.hr (I.B.); 2 Eberly College of Science, The Pennsylvania State University, University Park, State College, PA 16802, USA; 3 The Henry C. Lee College of Criminal Justice and Forensic Sciences, University of New Haven, West Haven, CT 06516, USA; 4 Medical School, University of Split, 21000 Split, Croatia; mmiskulin@me.com; 5 Faculty of Dental Medicine and Health, Josip Juraj Strossmayer University of Osijek, 31000 Osijek, Croatia; martina.smolic@mefos.hr; 6 Faculty of Medicine, Josip Juraj Strossmayer University of Osijek, 31000 Osijek, Croatia; 7 Medical School, University of Rijeka, 51000 Rijeka, Croatia; 8 Medical School REGIOMED, 96450 Coburg, Germany; 9 Medical School, University of Mostar, 88000 Mostar, Bosnia and Herzegovina; 10 Clinical Hospital “Sveti Duh”, 10000 Zagreb, Croatia; 11 Department of Nursing, University North, 48000 Varaždin, Croatia; 12 Department of Health Studies, University of Split, 21000 Split, Croatia; 13 Clinic for Traumatology, University Hospital “Sisters of Mercy”, 10000 Zagreb, Croatia; 14 Department of Orthopaedics and Traumatology, University Hospital Dubrava, 10000 Zagreb, Croatia; 15 Aksis Specialty Hospital, 10000 Zagreb, Croatia; 16 General Hospital Karlovac, 47000 Karlovac, Croatia; damir.cacic01@gmail.com

**Keywords:** knee osteoarthritis, guidelines, drug therapy, pharmacogenomics, intra-articular injections, mesenchymal stem cells

## Abstract

Osteoarthritis is the most common musculoskeletal progressive disease, with the knee as the most commonly affected joint in the human body. While several new medications are still under research, many symptomatic therapy options, such as analgesics (opioid and non-opioid), nonsteroid anti-inflammatory drugs, symptomatic slow-acting drugs in osteoarthritis, and preparations for topical administration, are being used, with a diverse clinical response and inconsistent conclusions across various professional societies guidelines. The concept of pharmacogenomic-guided therapy, which lies on principles of the right medication for the right patient in the right dose at the right time, can significantly increase the patient’s response to symptom relief therapy in knee osteoarthritis. Corticosteroid intra-articular injections and hyaluronic acid injections provoke numerous discussions and disagreements among different guidelines, even though they are currently used in daily clinical practice. Biological options, such as platelet-rich plasma and mesenchymal stem cell injections, have shown good results in the treatment of osteoarthritis symptoms, greatly increasing the patient’s quality of life, especially when combined with other therapeutic options. Non-inclusion of the latter therapies in the guidelines, and their inconsistent stance on numerous therapy options, requires larger and well-designed studies to examine the true effects of these therapies and update the existing guidelines.

## 1. Introduction

It is approximated that 250 million people worldwide suffer from osteoarthritis (OA), with an increasing trend in prevalence during the last decades, which continues to rise [1,2,3]. According to Global Burden of Diseases, Injuries, and Risk Factors Study 2015 by Disease and Injury Incidence and Prevalence Collaborators, approximately 85% of the burden of OA worldwide is connected with knee OA, with an estimated prevalence of 10% in men and 13% in women aged 60 and above [4,5]. OA is challenging to treat. The gold-standard end-stage therapy is total joint replacement surgery, without any effective therapeutic option available to stop OA from developing or progressing [1]. As a chronic disease with pain and diminished joint mobility and function as the dominant symptoms, pain management and lifestyle changes are the only available therapeutic option for low-grade OA. Therapeutic measures including intra-articular applications of corticosteroid injections, hyaluronic acid injections, platelet-rich plasma, or mesenchymal stem cells may slow down the existing condition according to some studies. Still, these results are often inconsistent, with different strengths of recommendation across different professional societies’ guidelines, as can be seen in Table 1 and Table 2 [6,7,8,9,10]. This comprehensive literature review aims to compare recent guidelines for the most-often-used pharmaceutical and biological treatment options and review the recent meta-analyses for potential new insights into these procedures.

## 2. Literature Search Methodology

To access the most recent literature with the highest level of evidence, a literature search of PubMed was provided using filters for systematic reviews and meta-analyses only, from 1 January 2018 until 10 February 2021. The term *knee osteoarthritis* was combined with the most commonly used pharmaceutical agents for its treatment using the commands AND and OR. The overall search included the following terms: (knee osteoarthritis) AND ((acetaminophen) OR (paracetamol) OR (opioids) OR (tramadol) OR (morphine) OR (oxycodone) OR (NSAID) OR (ibuprofen) OR (ketoprofen) OR (naproxen) OR (etoricoxib) OR (celecoxib) OR (rofecoxib) OR (DMOAD) OR (SADOA) OR (SYSADOA) OR (glucosamine) OR (chondroitin) OR (topical) OR (corticosteroid) OR (glucocorticoid) OR (methylprednisolone) OR (betamethasone) OR (triamcinolone) OR (dexamethasone) OR (hyaluronic acid) OR (hyaluronan) OR (platelet-rich plasma) OR (PRP) OR (mesenchymal stem cells) OR (MSC) OR (stromal vascular fraction) OR (SVF)). This search generated a total of 133 results, of which after reading the title and/or abstract, 42 papers satisfied the topic and the point of this article. These articles were read in full and included in the review. The 4 guidelines of well-known professional societies for the treatment of knee OA were included to compare the guidelines with the latest and most significant literature. The remaining 61 references were already known to the authors and/or were included in order to increase the quality of the work, improve the readability of the article itself, and write the introduction, the section on pharmacogenomics, and parts of the individual chapters’ conclusions.

## 3. Peroral Treatment

When OA becomes symptomatic, patients start to use some pharmacological agents, either recommended by the doctor or on their own. There is a wide range of agents used in treating symptomatic OA, from acetaminophen and nonsteroidal anti-inflammatory drugs (NSAIDs) to opioid analgesics and cartilage active agents.

### 3.1. Analgesics

#### 3.1.1. Acetaminophen (Paracetamol)

Acetaminophen is commonly used as a first-line analgesic in the treatment of various painful conditions. Despite its common use, the exact mechanism of acetaminophen action has not yet been established [11]. However, there are increasing doubts regarding the efficacy of acetaminophen in patients with OA [12]. While some researchers recommend acetaminophen as a very potent analgesic, meta-analyses report that acetaminophen in a maximal daily dose does not have a satisfactory effect in knee OA [13]. A recent Cochrane review and meta-analysis including 3541 patients with either hip or knee OA found no statistical difference of subjective pain intensity, physical function, or the observed side effects in the acetaminophen group compared to the placebo [14]. On the other hand, a network meta-analysis by Jung et al. showed that acetaminophen is clinically effective in knee OA patients with mild to moderate pain [15]. The American College of Rheumatology/Arthritis Foundation (ACR/AF) gave a conditional recommendation for acetaminophen use due to its small effect size when used as monotherapy, but it may be used for short-term or periodic use in patients who have a contraindication for other analgesic drugs [7].

It is important to emphasize that clinical improvement is the primary target of analgesic OA therapy; therefore, acetaminophen should not be dropped in these patients altogether but should instead be replaced by NSAIDs as first-line treatment in knee OA and reserved for situations in which they are contraindicated, which is in accordance with the Osteoarthritis Research Society International (OARSI) 2019 guidelines, which gave a conditional recommendation against their use [6]. The European Society for Clinical and Economic Aspects of Osteoporosis, Osteoarthritis and Musculoskeletal Diseases (ESCEO) 2019 guidelines gave a conditional recommendation for acetaminophen use only for short-term rescue analgesia in combination with long-term chondroitin sulfate or glucosamine (9). The American Academy of Orthopaedic Surgeons (AAOS) was not able to give a recommendation for or against the use of acetaminophen [8]. Acetaminophen should still be prescribed with caution because there are known side effects. In some patients, higher doses or prolonged use of acetaminophen can be hepatotoxic [12]. Comparing the safety profile of acetaminophen with NSAIDs, acetaminophen has fewer adverse effects, but the risk of liver toxicity cannot be neglected. For that reason, the Food and Drug Agency in 2011 issued a warning and communication to drug manufacturers to reduce the dose of acetaminophen in prescription drug products to 325 mg [16]. 

Concerns in relation to other possible adverse events associated with acetaminophen use have been raised. One observational study found that using high doses of acetaminophen (>3 g/day) is associated with a higher risk of hospitalization due to gastrointestinal bleeding compared to doses of 3 g/day or lesser [17]. Other studies indicated a decreased glomerular filtration rate with prolonged acetaminophen use of daily doses above 3 g and a higher incidence of hypertension [18]. Such concerns have to be acknowledged, but it also needs to be kept in mind that most of the studies reporting these adverse events were observational studies. In addition, acetaminophen was commonly prescribed in the elderly due to their comorbidities and higher susceptibility to NSAID-caused adverse events, thereby creating allocation bias [19].

#### 3.1.2. Opioids

Opioids (tramadol, morphine, oxycodone, etc.) are not readily prescribed in the treatment of OA. Opioid analgesics are agonists of the opioid receptors in the central nervous system (CNS), whose activation leads to CNS depression [20]. They have a well-known side-effect profile, including constipation, nausea, and vomiting, in addition to their very high addiction potential. In a direct comparison to NSAIDs, tramadol was shown to be inferior at short-term (4–12 weeks) physical function improvement and tolerability for neuropathic, low-back, and OA pain [21]. Opioids are generally indicated for short-term OA therapy in patients where other analgesics are unsuccessful or contraindicated for any reason [22]. They are also a good choice in patients who are not candidates for joint replacement [7,9]. The recommendations of professional guidelines differ on this topic. The AAOS gave a positive recommendation for the use of tramadol in the symptomatic treatment of knee OA; however, it found evidence of the use of other opioids or transdermal patches inconclusive [8]. The ACR/AF gave a conditional recommendation for the use of tramadol, while other opioid analgesics were given a conditional recommendation against use, indicating both should be used only when other therapeutic options have been exhausted [7]. ESCEO guidelines have a similar stance, giving a conditional recommendation for the use of opioids as a third-line therapy option prior to knee replacement surgery when other pharmacological options (including intra-articular corticosteroids and hyaluronic acid (HA)) are unsuccessful in symptomatic relief [9]. The only guideline that gave a negative recommendation was that by OARSI. A strong recommendation against the use of oral or transdermal opioids for OA treatment was given due to their high addiction potential and limited efficacy [6]. 

According to a Cochrane review, tramadol alone or in combination with acetaminophen had no significant benefit on mean pain or function in patients with OA compared to the placebo [23]. A systematic review and meta-analysis that investigated opioid usage for OA pain found low tolerability of opioids, without clinically relevant efficacy in controlled studies from 4 to 24 weeks for OA pain [24]. Similar findings were reported in a recent meta-analysis by Osani et al. The authors concluded that opioids showed minor benefits on pain and function compared with the placebo from 2 to 12 weeks of treatment, which did not improve the patients’ quality of life. Furthermore, the authors indicated that stronger opioids (morphine, oxycodone) displayed inferior clinical results than weak/intermediate opioids (codeine, tramadol) but also increased the risk of experiencing more adverse effects [25]. These latest findings weigh in favor of the negative recommendation given by most guidelines, in our opinion; however, a rational approach on a patient-to-patient basis should be taken to identify the need for opioid therapy where other options have failed, much like the three-step approach recommended by ESCEO.

### 3.2. Nonsteroidal Anti-Inflammatory Drugs (NSAIDs)

NSAIDs include two groups of drugs: non-selective cyclooxygenase (COX) inhibitors and selective cyclooxygenase-2 (COX-2) inhibitors, such as etoricoxib and celecoxib. They have an analgesic and anti-inflammatory effect. Because of their anti-inflammatory effect, they have good efficacy in the treatment of OA-related pain. Nevertheless, these drugs should be used very carefully because of their side-effect profile in chronic use, especially gastrointestinal and cardiovascular effects [26,27,28]. Gastrointestinal side effects are more likely to occur in patients with some risk factors such as age over 60, high NSAID doses, long therapy duration, co-administration of two or more NSAIDs, and Helicobacter pylori infection [29]. In the cases where this risk is increased, non-selective COX inhibitors in combination with a proton pump inhibitor or selective COX-2 inhibitors should be administered [30]. A study by Nissen et al. investigated the cardiovascular safety of celecoxib, a selective COX-2 inhibitor, and non-selective COX inhibitors (naproxen, ibuprofen). Non-significant differences in the risk of a cardiovascular event were observed between the drugs, but celecoxib showed significantly lower rates of gastrointestinal events than non-selective COX inhibitors and also lower rates of renal side effects compared to ibuprofen [31]. In a systematic review and network meta-analysis of long-term (≥12 months) trials by Gregori et al., celecoxib was the only NSAID associated with improvements in pain, but the association was small and without observed improvements in physical function [32]. Given only the minor or no clinical benefits of long-term NSAID use and considering the possible risk of adverse effects, NSAID therapy should be restricted only to short-term treatment. Different conclusions have been drawn regarding the most potent NSAID. A meta-analysis by da Costa et al. indicated that oral use of diclofenac 150 mg/day is the most effective for pain management and physical function improvement compared to other NSAIDs such as rofecoxib, lumiracoxib, etoricoxib, celecoxib, ibuprofen, and naproxen [33]. Of all the available NSAIDs, naproxen was found to be the most effective in both symptom relief and positive functional outcomes in a network meta-analysis, which included all randomized control trials in the English language until 2015, that compared the clinical effectiveness of available oral and intra-articular pharmacologic agents (NSAIDs, acetaminophen, corticosteroids, and hyaluronic acid) to each other and to the placebo [34]. The observed results were even stronger when oral naproxen was used with intra-articular corticosteroid application. OARSI, ESCEO, and ACR/AF guidelines agree on the recommendation of oral NSAIDs as first-line short-term therapy for persistent pain in OA patients who are not at high risk for a cardiovascular event [6,7,9,10]. The AAOS gave a positive recommendation for the use of NSAIDs in the symptomatic treatment of knee OA as first-line therapy [8].

The positive results of NSAID therapy are of no surprise from a pathophysiologic point of view, as the key driver of OA progression is a low-grade chronic inflammation caused by an imbalance between anabolic and catabolic processes of the articular osteochondral unit [35]. 

### 3.3. Symptomatic Slow-Acting Drugs in Osteoarthritis (SYSADOA)

According to Steinmeyer and co-authors, glucosamine and chondroitin sulfate are in a group of symptomatic slow-acting drugs in osteoarthritis (SYSADOA) [29]. Glucosamine is a metabolic precursor of glycosaminoglycans, which are the components of the cartilage extracellular matrix (ECM), and chondroitin sulfate is a natural component of the ECM [35,36]. Evidence of the positive effects of glucosamine and chondroitin sulfate is still a matter of debate. Official guidelines have different attitudes toward the use of glucosamine and chondroitin sulfate in the treatment of knee OA. The AAOS, in its 2013 guidelines, does not recommend the use of glucosamine and chondroitin for patients with symptomatic knee OA, with a strong strength of recommendation [8]. OARSI gave recommendations for the symptom relief effect and disease-modifying effect for both the drugs separately in its 2014 guidelines but did not include them in its 2019 knee OA guidelines [6,37]. The recommendation for the symptom relief effect was uncertain and for the disease-modifying effect was not appropriate. The main reason for the recommendation was the drug’s weak effect and very heterogeneous results between studies [37]. Glucosamine may be used in patients with NSAID intolerance or patients with high gastrointestinal and cardiovascular risk. Although their effect in symptomatic relief of patients with knee OA cannot be denied, ACR/AF guidelines gave a strong recommendation against the use of glucosamine and chondroitin sulfate due to discrepancies in analyzed studies, which indicated a possible publication bias, high placebo effect, and unknown biological mechanisms of their effect [7]. 

However, recent meta-analyses indicate the potential benefits of therapy with SYSADOA in patients with knee OA. A systematic review and network meta-analysis of long-term (≥12 months) trials found that glucosamine sulfate is related to pain reduction but also improvements in physical function and joint structure [32]. Another meta-analysis concluded that supplementation with glucosamine or chondroitin sulfate reduces pain levels measured by the visual analog scale (VAS) in knee OA patients but do not improve the Western Ontario and McMaster Universities Osteoarthritis Index (WOMAC) score for pain, function, or stiffness [38]. Zhu et al. noticed superior benefits of chondroitin in alleviating pain and improving physical function compared with the placebo and also the role of glucosamine in reducing joint stiffness. They also emphasized a good safety profile and great tolerance of the aforementioned supplements [39]. Different conclusions regarding the efficacy of SYSADOA may be a consequence of various qualities of glucosamine and chondroitin preparations in numerous studies. This idea was corroborated by a recent meta-analysis that marked that prescription-grade chondroitin sulfate and prescription-grade crystalline glucosamine sulfate are more effective in reducing pain in knee OA than nutraceutical grade or over-the-counter (OTC) glucosamine or chondroitin preparations [40]. Similar conclusions were made by Honvo and colleagues, who indicated that prescription-grade preparations with chondroitin sulfate achieve better results for pain and functional status [41]. ESCEO guidelines recommend only pharmaceutical-grade chondroitin sulfate and crystalline glucosamine as first-line long-term therapy in symptomatic knee OA as both single therapy and in combination with acetaminophen, distinguishing them from the same products of weak pharmaceutical quality. They do not recommend the use of over-the-counter products containing both chondroitin sulfate and glucosamine [9]. 

Given the fact that new research does not dismiss SYSADOA as a potential symptomatic therapy for knee OA and that the guidelines do not unequivocally advise against their use, larger placebo-controlled studies with prescription-grade preparations are needed to re-evaluate current guidelines and draw stronger conclusions.

### 3.4. Pharmacologic Treatment in the Pharmacogenomic Context

New pharmacogenomic research indicates that the often-observed inter-individual differences, based on the patient’s genetic make-up, should be taken into consideration when prescribing pharmacologic treatment. This is emphasized with reports of up to 50% of patients using an analgesic treatment who do not experience adequate pain relief and with pain being one of the leading symptoms of OA that can predispose the patients to develop depression if it is not adequately addressed and treated [42,43]. The field of pharmacogenomics aims to identify the genetic markers responsible for variable patient drug responses by looking at the genotype of drug-metabolizing enzymes, transporter proteins, target receptors, and others in order to determine the most effective and safest medication and its dose on a case-to-case basis, in contrast to the currently used one-size-fits-all approach used in clinical practice today. The key information pharmacogenomics brings to the clinician is the net result of different allele combinations, referred to as the enzyme phenotype, which defines its function as reduced, normal, or increased [44].

It should be stated that knowing the genetic profile alone is not enough to completely alleviate pain in patients suffering from musculoskeletal pain. Other factors such as the environment, age, sex, previous medical conditions, and lifestyle greatly contribute to the individual sensation of pain [45]. However, pharmacogenomic research offers a new perspective on some of the most commonly used analgesics to treat OA, such as NSAIDs and opioids. 

#### 3.4.1. NSAIDs 

The different bioavailability based on the CYP2C8 (a member of the cytochrome P450 family) genotype is shown to play a role in patients developing potentially serious adverse drug reactions with prolonged use of NSAIDs, such as gastrointestinal or cardiovascular events [46]. Single-nucleotide polymorphisms for another member of the cytochrome P450 enzyme family, CYP2C9, have been found to influence the metabolism rate of celecoxib and flurbiprofen. For patients who have a determined poor metabolizer phenotype (CYP2C9 *3/*3), a 50% reduction in the starting dose is suggested to avoid potential side effects; however, it is not part of any official guideline [47]. Another study found an increased risk of gastrointestinal tract bleeding in patients carrying CYP2C8*3 and CYP2C9*2 alleles when using NSAIDs that are the substrate of both of these enzymes, such as ibuprofen and diclofenac [48].

#### 3.4.2. Opioids

Although not commonly prescribed for OA patients, opioid analgesics are a group of drugs most commonly associated with genetic polymorphisms. Tramadol, codeine, and oxycodone are all metabolized by CYP2D6 in the liver and bind to the opioid receptor, both of which have demonstrated the ability to impact the effects and side-effect profile of the drugs [49]. Another enzyme linked to the effect of opioid analgesics is catechol-O-methyltransferase, which degrades endogenous catecholamines. Its polymorphisms affect the analgesic efficacy of an opioid drug [49].

Detailed clinical guidelines are available for the interpretation of pharmacogenomic results based on the CYP2D6 genotype, whilst a focused review of the opioid receptor M1 subunit (OPRM1) and COMT polymorphisms did not produce any therapeutic dosing recommendation due to mixed and insufficient evidence of a clinically relevant effect [50]. 

The implementation of pharmacogenomic results in daily clinical practice is a challenge because it requires an interdisciplinary team of physicians. However, in the future, with the development of more robust genetic screening platforms and increased numbers of patients willing to test themselves for their unique polymorphisms, new tools should be made available to ease the interpretation of data in a reliable, easy-to-understand, and fast manner, possibly using the advantages of artificial intelligence [51].

## 4. Topical Treatment

- Topical NSAIDs

Topically used NSAIDs (diclofenac, ketoprofen, and ibuprofen) are a very simple and popular method in the therapy of OA. Their main advantage compared to oral NSAIDs is their side-effect profile, which is greatly reduced, with only 5–15% serum concentration compared to that of oral administration [29]. The most common side effects of topical NSAIDs include skin reactions on the application site, including dermatitis, pruritus, and rash, while systemic gastrointestinal and cardiovascular side effects are rare and less common than after oral use [52]. A Cochrane review showed that gastrointestinal side effects during topical application of NSAIDs are the same as in the placebo group [53]. The effectiveness of topical administration is also very good. According to the same Cochrane review, after topical application of diclofenac or ketoprofen, 60% of patients reported pain reduction by 50% [53]. However, a systematic review by Concoff et al. demonstrated that topical NSAIDs have smaller effect estimates than acetaminophen, intra-articular corticosteroids, and intra-articular hyaluronic acid regardless of molecular weight [54]. Knowing that OA is a condition predominantly affecting the older population, who is at higher risk of experiencing the side effects of prolonged NSAID use due to either underlying medical conditions or polypharmacy potentiating that effect, the reduced side-effect potential is welcomed in this population [55,56]. However, caution should be employed when co-administering oral and topical NSAIDs, particularly in patients who have previously experienced NSAID-related side effects [57]. Guidelines are in unison in their positive recommendation of topical NSAID therapy. The AAOS gave a positive recommendation for the use of topical NSAIDs in the symptomatic treatment of knee OA [8]. OARSI guidelines recommend topical NSAIDs as first-line treatment for pain relief in knee OA, while the ACR/AF gave a strong recommendation for their use and suggested they be used before oral NSAIDs [6,7]. ESCEO guidelines recommend topical NSAIDs to be used before oral NSAIDs when optimal pain relief is not achieved by first-line SYSADOA and acetaminophen [9].

## 5. Intra-Articular Injections

### 5.1. Corticosteroid Injections

Corticosteroids are a well-known group of drugs used to treat various inflammatory conditions in almost every field of medicine. Physiologically, they are stress hormones that bind to the glucocorticoid receptor and regulate multiple processes throughout the body by modifying gene expression [58]. Injected corticosteroids treat a targeted location, such as inflammation or pain caused by tendinitis or pain in the osteoarthritic joint.

A Cochrane review of intra-articular corticosteroid injections concluded that corticosteroids could offer moderate pain relief and a little improvement in physical function. Intra-articular corticosteroids were shown to have a similar side-effect profile compared to the placebo. The quality of evidence, however, was considered to be very low for all results, since the analyzed study results were largely inconsistent and the evidence was based on many small studies of poor quality [59].

Even though intra-articular glucocorticoid injections are widely used in clinical practice and have some effect in short-term joint pain improvement, studies are showing its inferiority at 1 year after administration compared to physical therapy [60].

Although both methods show positive results regarding pain management and function improvement in patients with knee OA, treatment with intra-articular steroids before physical therapy is not associated with additional benefits [61]. A study by O’Neill et al. showed that corticosteroid injection into knee joints with magnetic resonance imaging (MRI)-confirmed synovial thickening significantly reduces synovial tissue volume, which is correlated with pain reduction [62]. In addition, with the corticosteroid effect wearing off, an increase in both synovial tissue volume and pain recurrence was observed, indicating the potential of repetitive treatment with intra-articular steroids for patients with confirmed synovial inflammation. These results were reinforced by the findings of McCabe et al., who investigated the relationship between synovial fluid blood cell count and response to therapy with intra-articular steroids, concluding that pain reduction is greater in patients with a higher synovial white blood cell count [63].

However, intermittent injections of corticosteroids were not associated with long-term pain reduction in a systematic review and network meta-analysis of long-term (≥12 months) trials by Gregori et al. [32]. Still, corticoids were the only intra-articular therapy option (among hyaluronic acid and PRP injections) that had a statistically significant effect on reducing pain compared to the intra-articular placebo according to Jevsevar et al. [34]. The same study ranked intra-articular corticosteroids as the most promising therapy option in reducing pain, with oral NSAIDs and other intra-articular options falling behind. Although intra-articular corticosteroids are widely used as a short-term pain relief therapy option, Saltychev et al. analyzed the magnitude and duration of their effect on pain severity in knee OA. They reported mild to moderate pain reduction for up to 3 months after the initial injection of corticosteroids. Results between corticosteroids differed from a strong effect with betamethasone to statistically insignificant effects with triamcinolone [64]. Nevertheless, a recent network meta-analysis claimed that extended-release corticosteroids (triamcinolone acetonide extended-release injectable suspension) may provide an additional clinical benefit over standard-release corticosteroids (triamcinolone, betamethasone, hydrocortisone, methylprednisolone, and cortisone), but indicated the need for further research comparing the two forms of corticosteroid injections with the placebo [65]. 

The guidelines again differ in their recommendation of intra-articular corticosteroid therapy. ESCEO gave a weak recommendation for corticosteroids, only to be used when patients have a contraindication for the use of NSAIDs or have insufficient relief on NSAID therapy, for short-term pain relief, suggesting also that a greater effect may be expected in patients with higher pain intensity [9]. OARSI gave a conditional recommendation for the use of intra-articular corticosteroids for short-term pain relief, with a good clinical practice statement indicating an acceptable safety profile for patients with comorbidities [6]. The ACR/AF gave a strong recommendation for the use of intra-articular glucocorticoid injections for short-term pain relief [7]. The AAOS was not able to give a recommendation for or against the use of intra-articular corticosteroids in its 2013 guidelines [8]. Guideline discrepancies should be considered when deciding on intra-articular corticosteroid therapy, bearing in mind its chondrotoxic effect [66,67]. According to the available body of evidence, intra-articular corticosteroids should be reserved for persistent pain in higher-grade OA, as most guidelines agree, perhaps using other intra-articular options for short-term pain treatment in younger individuals and those with low-grade OA.

### 5.2. Viscosupplementation (Hyaluronic Acid)

Hyaluronic acid (HA) is a molecule from the group of glycosaminoglycans. HA properties vary based on its molecular weight and molecular structure, thus making it a heterogeneous group of compounds rather than a single molecule. The main roles of HA are lubrication of the joint and chondroprotection from mechanical damage [68]. Intra-articular HA injections have an anti-inflammatory, mechanical, and analgesic effect and also a positive effect on proteoglycan and glycosaminoglycan synthesis [69]. Intra-articular HA application is a safe procedure, with only an increased risk of nonserious, transient local reactions reported, as reported in a systematic review and meta-analysis involving more than 8000 patients by Miller and colleagues [70]. In a systematic review by Altman et al., repeated HA injections resulted in the retention or improvement of the positive effects on knee pain, without increased safety risk, stressing the safety of repeated HA injections as one of its advantages [68].

The quality of HA products has been improving in recent years. Thus, high-molecular-weight HA (HMWHA) emerged, which was believed to have a better effect on the joint than low-molecular-weight HA (LMWHA) [69]. This idea was confirmed by a systematic review that showed a greater effect of hyaluronic acid compared to non-selective NSAIDs and selective COX-2 inhibitors, but only when higher-molecular-weight hyaluronic acid was used for the treatment of knee OA [54]. A systematic review by Altman and colleagues studied the anti-inflammatory properties of intra-articular hyaluronic acid and found that, in contrast to LMWHA, HMWHA possesses not only multivalent sites for CD44 binding but also interacts with toll-like receptor (TLR) and intercellular adhesion molecule-1 (ICAM-1) receptor signaling [71]. Using these mechanisms, HMWHA can downregulate the expression of proinflammatory cytokines, matrix metalloproteinases, prostaglandins, and nitric oxide, molecules responsible for joint inflammation through complex pathophysiologic mechanisms [35]. OARSI and ACR/AF guidelines do not comment on different molecular weights of HA [6,7]. AAOS guidelines state that there are no observed differences for substances over 750 kDa, but HMWHA did show superiority over LMWHA in the studies it analyzed [8]. ESCEO guidelines also commented that the analyzed studies did show the inferiority of LMWHA and that cross-linked HMWHA is associated with a higher occurrence of adverse events [9]. These observations and comments were not included in the final recommendation of these guidelines [8,9].

According to a study by Bowman et al., there are some groups of patients who are more likely to have better outcomes after hyaluronic injection treatment [72]. These are patients with mild to moderate OA, patients older than 60 with moderate OA, and patients who had a positive response to the first injection. According to the same study, patients who respond positively are less likely to undergo knee replacement. Still, Gregori et al. reported no association of hyaluronic acid with long-term pain improvement in patients with knee OA [32].

Although the AAOS could not recommend HA usage for patients with symptomatic knee OA, OARSI gave a conditional recommendation for the use of intra-articular HA for effects over 12 weeks after application, with a good clinical practice statement for patients with comorbidities, while also indicating an acceptable and more favorable safety profile than repeated corticosteroid injections [6,8]. The ACR/AF gave a conditional recommendation against the use of HA in OA, due to a low symptom relief effect when compared to the placebo in studies with a low risk of bias [7]. ESCEO gave a weak recommendation for HA, only to be used when patients have a contraindication for the use of NSAIDs or have insufficient pain relief on NSAID therapy [9].

A systematic review and meta-analysis by Miller et al. concluded that intra-articular application of hyaluronic acid to the knee joint provides statistically significant, but not clinically important, improvements in pain and knee function, but with a lower risk of side effects compared to orally administered NSAIDs, which are positively recommended by all professional societies’ guidelines included in this article [73]. As the guidelines are inconsistent regarding the use of HA in the treatment of knee OA, future research should focus on patient inclusion criteria, particularly to the OA stage and pain levels. Bowman et al. concluded that the application of hyaluronic acid has more effect when therapy is carried out in patients with moderate pain [72]. On the same track were the results of Nicholls and co-workers that demonstrated that intra-articular application of HA, in comparison with the placebo, leads to significant pain reduction in patients with early to moderate OA compared to when the same therapy is administered to patients with end-stage OA [74]. The inclusion of a different patient profile in the studies, with different stages of OA, together with inconsistent HA properties (molecular weight and structure) across studies, can lead to deceptive results and erroneous conclusions regarding the effect of HA therapy.

### 5.3. Biological Treatment

#### 5.3.1. Platelet-Rich Plasma

Defined as a volume of plasma with a platelet concentration several times higher than in peripheral blood, platelet-rich plasma (PRP) exerts its effect by locally releasing chemokines, cytokines, growth factors, adhesive proteins, proteases, and other small molecules. Based on the leukocyte and fibrin content, there are four general categories of PRP: leukocyte-rich PRP (L-PRP), leukocyte-reduced PRP (P-PRP), leukocyte platelet-rich fibrin, and pure platelet-rich fibrin [75]. Studies generally agree on the short- and medium-term analgesic effect of PRP in knee OA; however, it is difficult to draw strict conclusions regarding clinical results due to different modes of PRP preparation and application [76,77]. A recent literature review and meta-analysis including 33 studies on the effect of PRP in OA demonstrated significant positive differences in the VAS, WOMAC, Knee Osteoarthritis Outcome Score (KOOS), and International Knee Documentation Committee (IKDC) scales when compared to HA and the placebo, while the VAS difference was not significant when compared to corticosteroids. In pooled estimates, there was no statistically significant difference noted for adverse events of PRP therapy compared to the control group (placebo, HA, corticosteroids, and mesenchymal stem cells). Multiple injections were also shown to be superior to a single injection, but this effect was only observed when three injections were applied [78]. Similar results regarding the frequency of PRP injections were shown in a meta-analysis by Vilchez-Cavazos and colleagues, where no difference in pain improvement was observed for single versus multiple PRP injections; however, there was a significant difference in functional outcomes at 6 months’ follow-up for a triple versus a single injection [79].

These results are further reinforced by a Bayesian network meta-analysis of 30 studies that demonstrated the superiority of PRP to HA, placebo, and corticosteroid injection for VAS and WOMAC scores at 3, 6, and 12 months’ follow-up [80]. Two meta-analyses, of 12 and 10 studies, respectively, comparing the effects of PRP and HA, found that patients in the PRP group showed a statistically significant difference in pain reduction (measured by VAS and WOMAC pain scales) at 6 and 12 months’ follow-up, while there was no observed difference for clinical outcomes measured by KOOS and other WOMAC scales [76,81]. Meta-analyses, including 20 and 15 studies respectively, comparing PRP to HA by Tang et al. and Han et al. demonstrated a positive effect for both pain and function scores, and a meta-analysis by Zhang et al. reported an improvement in the WOMAC function score at 12 months’ follow-up, while there was no significant difference between methods at 6 months after the treatment [82,83,84]. A meta-analysis by Chen et al. found that WOMAC total scores superiorly improved in patients treated with PRP compared with patients treated with HA [85]. All of the conducted meta-analyses had a common result of statistically significant pain reduction after PRP therapy compared to other intra-articular drugs commonly used, in contrast to functional patient outcomes that have not been consistently reported. This leads to a conclusion that PRP may be the best option for patients who present with pain as the leading symptom for short- to middle-term therapeutic benefit and for patients who present at an earlier stage of OA with mild symptoms [86]. The effect of PRP combined with various other preparations or procedures is an interesting area of research that includes combinations of PRP with stem cells or HA. A recent study observed the effect of treatment with either a single PRP injection or a combination of PRP and hyaluronic acid injection in 78 patients with Kellgren–Lawrence stage 2 OA [87]. It demonstrated that patients achieved better pain relief at 1-month follow-up with a single injection, while the combination group had greater VAS reduction at 6 months’ follow-up. There were no other differences between the two groups, indicating that the combined approach could be the method of choice for long-term pain relief in OA patients [87]. A meta-analysis by Zhao et al. demonstrated the greater benefit of combined PRP and HA injection compared to single therapy for both pain scores at 6 months’ follow-up and function at 12 months’ follow-up [88]. Superior benefits of the combined therapy were corroborated in a systematic review and meta-analysis by Karasavvidis et al., who concluded that patients treated with a combination of PRP and HA had better clinical results for both pain and function (measured by VAS at 3, 6, and 12 months’ follow-ups and 12-month WOMAC physical function and stiffness score) compared to patients treated with HA only [89]. 

The possible therapeutic potential of PRP products in OA is not fully investigated and used, and due to the heterogeneity of study methods with a high risk of bias, the ACR/AF and OARSI guidelines strongly recommend against its use before these problems are resolved in further studies [6,7]. The AAOS was not able to give a recommendation for or against the use of PRP in its guidelines, and ESCEO did not include PRP in its guidelines [8,9]. A recent systematic review and meta-analysis by Belk et al. was one of many studies demonstrating undeniable clinical improvements of PRP treatment, but it also discussed the leukocyte content in PRP injections. Although having a higher concentration of growth factors, leukocyte-rich PRP has more proinflammatory properties than leukocyte-poor PRP, indicating the need for further research and product standardization [90]. Even though numerous studies with a high level of evidence show excellent clinical improvements in patients with knee OA treated with intra-articular PRP injections, product characterization and dosage, as well as proper timing, treatment repetition period, and application technique, need to be standardized for guidelines to consider including PRP in OA treatment protocols.

#### 5.3.2. Mesenchymal Stem Cells (MSCs)

Articular cartilage, the main affected tissue in OA, has a limited capacity for self-renewal. Since OA is a complex pathophysiological entity involving the whole joint, research efforts have been made to identify key regulating factors that could be used in the pharmacologic treatment of OA [35]. Because of their in vitro ability to differentiate into a variety of cell types and their regenerative and immunoregulatory properties, MSCs have attracted great interest in OA treatment. The persistence of mesenchymal stem cells was first demonstrated in the bone marrow, after which their existence was also confirmed in other tissues such as fat, peripheral blood, placental tissue, umbilical cord, synovial tissue, and dental pulp [91]. Autologous bone-marrow-derived MSCs (BM-MSCs) and adipose-derived MSCs (AD-MSCs), also frequently called adipose-derived stromal vascular fraction (AD-SVF), are currently predominantly used for the treatment of knee OA (previously cultured or directly isolated and applied), while other cell sources such as synovial or allogeneic placental tissue require more testing to enter everyday clinical practice [92,93].

In the natural course of OA, intra-articularly applied MSCs accumulate in joints and adjacent bone marrow lesions, suggesting their role in the response to joint injury, but the mechanism by which stem cell therapy may be effective in OA remains unclear [94,95]. Nevertheless, MSCs are increasingly used in clinical practice, with reports of their benefits regarding symptom relief and joint functionality [96,97,98]. However, a strong recommendation against the use of MSCs has been made in the ACR/AF and OARSI guidelines due to the various methodology (discrepancies in tissue origins of MSCs, cell numbers, and culture methods) and application strategies used in clinical studies that may influence therapeutic effects and, therefore, the clinical response [6,7]. MSC therapy was not included in AAOS and ESCEO guidelines [8,9].

Despite the negative recommendation by the key opinion makers, a number of clinical and scientific efforts have been made in the research on MSCs in OA treatment in the past 10 years. A meta-analysis that included five randomized controlled trials (four with BM-MSCs and one with AD-SVF) with 220 patients found a statistically significant reduction in pain intensity analyzed by the VAS and the Lysholm scale, but no difference in WOMAC. Functional outcomes analyzed by Lysholm and WOMAC scores demonstrated a significant improvement with a standard mean difference of 0.53%. This analysis also indicated that there were no differences in cartilage repair on an MRI examination [99]. Another meta-analysis looked at randomized controlled trials (RCTs) examining culture-expanded MSCs in OA treatment. It included a total of six studies (four with BM-MSCs, one with AD-MSCs, and one with placenta-derived MSCs) and 203 patients and reported a statistically significant reduction in pain symptoms measured by both the VAS and WOMAC. However, it also did not find any significant difference in cartilage repair based on MRI analysis or the whole-organ magnetic resonance score (WORMS) [100]. Another meta-analysis by Ma et al. looked at 10 RCTs (4 with BM-MSCs, 3 with AD-MSCs, 1 with adipose-derived mesenchymal progenitor cells (AD-MPCs), 1 with umbilical cord MSCs, and 1 with placenta-derived MSCs), excluding studies where there was a surgical intervention additional to MSC application. Their results demonstrated a significant reduction in perceived pain by the VAS and WOMAC and better stiffness, functionality, and total WOMAC scores for patients randomized to MSC treatment compared to the controls. They also reported increased cartilage volume in the MSC group; however, there was no significant difference in WORMS [101]. These observations were further reinforced in another meta-analysis including 19 studies (15 RCTs, 2 retrospective studies, and 2 cohort studies, of which 9 studies were with AD-MSCs, 5 with BM-MSCs, peripheral blood stem cells in 1 study, and MSCs from a fetus in 4 studies) that found statistically significant pain relief effectiveness measured by the VAS at 12 months’ and KOOS and WOMAC at 6 months’ follow-up. The included studies demonstrated no side-effects of intra-articular MSC therapy [102]. In a systematic review and meta-analysis by Maheshwer and colleagues including 25 studies, a different result was observed, as demonstrated by no significant pain improvement, but a functional and cartilage volume improvement (0.66 and 0.84 standardized mean difference (SMD), respectively) [103]. They did, however, note that the observed cartilage quality did not reach statistical significance in the analyzed studies. The studies analyzed included different origins of mesenchymal stem cells, such as synovial tissue (1 study), bone marrow aspirate (8 studies), adipose tissue (14 studies), peripheral blood (1 study), and human umbilical cord blood (1 study). The potential of bias in the analyzed studies was high with 17 of 25 analyzed studies being graded as poor or fair [103]. A broader systematic review including 17 studies (6 RCTs) using adipose (6 studies with AD-SVF, 2 with AD-MSCs), bone marrow (8 studies), and umbilical-cord-blood-derived MSCs (1 study) offered the same conclusions in terms of patient-reported pain and functionality outcome, with 15 of 17 included studies reporting this outcome. Regarding cartilage repair, the results differed as 9 of 11 studies reported improved cartilage state on MRI and 6 of 7 on a second-look arthroscopy [104]. A systematic review by di Matteo and colleagues including 23 studies (10 studies used a bone marrow aspirate concentrate and 13 studies used AD-SVF) assessed the studies by analyzing minimally manipulated mesenchymal stem cells and found a significant short-term benefit observed as an improvement in both pain and functional scores analyzed [105]. Follow-up times of included studies ranged from 6 to 34 months for stromal vascular fraction (SVF) studies and 24 days to 24 months for the bone marrow aspirate concentrate (BMAC). An included study had a follow-up of 8 to 16 years, but its design was different from the other included studies as it observed patients with osteonecrosis secondary to corticosteroid use. They also found no significant side effects associated with MSC application. The methodology of the analyzed studies was flawed as it included various adjuvant therapies to SVF, such as PRP or HA, and also different methods of administration, therefore skewing the exact effect of SVF on the analyzed outcomes and cartilage repair. Even though it did not offer any recommendations as it demonstrated a lack of high-quality studies or a straight clinical protocol being used, their study pointed out the short-term benefits of MSC therapy [105]. These studies reinforce the current evidence of the short-term benefits of MSC therapy for knee OA, with a side-effect profile that allows regular clinical intervention. We believe it is important to emphasize that the conducted meta-analyses and systematic reviews did report a high risk of bias in the examined studies and inconsistencies in study protocols. Problems associated with MSC therapy include dosing, harvest site, the number of delivered MSCs, and the characterization of delivered cell populations, as there is no standard procedure that can answer these questions. Proper product characterization is a step in the right direction for these procedures and should be performed to compare the MSC application techniques delivered [106]. Therefore, we believe the future of MSC research and therapy is to provide a method that is available to address these concerns and demonstrate clinical effectiveness in a large multicentric RCT.

## 6. Conclusions

Non-operative OA treatment is an ever-growing research field with a common goal of finding both the best symptomatic treatment and a disease-modifying treatment that would slow down or altogether stop further development of OA. In clinical practice, patients who present with OA are most commonly of older age, at which other comorbidities are a factor that has to be included in the individual treatment algorithm, therefore making it increasingly difficult to form universally applying treatment guidelines [107]. The guideline development process includes thorough literature reviews and a general consensus among physicians; therefore, discrepancies among guidelines are always expected. However, we believe that a more frequent guideline revision protocol should be implemented as the research pace in the field is great. In addition, the guidelines do not differentiate between the treatment of early and late OA. Updating the guidelines in this sense could have a positive effect in terms of slowing the course of the disease in many patients who have been diagnosed with OA at an early stage, thus significantly reducing the degree of disability as a consequence of late-stage OA. Furthermore, the research design should focus on providing answers to questions posed in the guideline development process, such as the heterogeneity of PRP and MSC procedures. New information gathered using this method would provide better-quality evidence necessary to establish better treatment protocols for knee OA.

## Figures and Tables

**Table 1:** Guideline recommendations for most commonly used oral and topical pharmacological agents in osteoarthritis treatment.

Guideline Author	Year of Issue	Acetaminophen	Opioid Analgesics		Peroral NSAIDs	SYSADOA	Topical NSAIDs
			Tramadol	Other			
AAOS	2013	Unable to give any recommendation	Positive recommendation	Inconclusive	Positive recommendation	Strong recommendation against use	Positive recommendation
ACR/AF	2020	Conditional recommendation for	Conditional recommendation for	Conditional recommendation against	Recommended as first-line treatment	Strong recommendation against use	Strong recommendation for use prior to oral NSAIDs
OARSI	2019	Conditional recommendation against	Strong recommendation against		Recommended as first-line treatment	Not included	Recommended as first-line treatment
ESCEO	2019	Weak recommendation against as single therapy, should be used as rescue medicine in addition to first-line treatment with SYSADOA	Conditional recommendation for as third-line treatment		Recommended as first-line, short-term treatment	Recommended as first-line, long-term treatment for pharmaceutical-grade products	Recommended in addition to SYSADOA and acetaminophen prior to oral NSAIDs

AAOS—American Academy of Orthopedic Surgeons; ACR/AF—American College of Rheumatology/Arthritis Foundation; OARSI—Osteoarthritis Research Society International; ESCEO—European Society for Clinical and Economic Aspects of Osteoporosis, Osteoarthritis and Musculoskeletal Diseases; NSAIDs—nonsteroidal anti-inflammatory drugs; SYSADOA—systemic slow-acting drugs in osteoarthritis.

**Table 2:** Guideline recommendations for most commonly used intra-articular options in osteoarthritis treatment.

Guideline Author	Year of Issue	IACS	IAHA	PRP	MSCs
AAOS	2013	Unable to give any recommendation	Not recommended	Unable to give any recommendation	Not included
ACR/AF	2020	Strong recommendation for short-term analgesia	Conditional recommendation against	Strong recommendation against, (heterogeneous studies, lack of preparation and application standardization)	Strong recommendation against (heterogeneous studies, lack of preparation and application standardization)
OARSI	2019	Conditional recommendation for short-term analgesia	Conditional recommendation for a long-term effect where multiple IACS are contraindicated	Strong recommendation against (non-standardized formulations, low-quality evidence)	Strong recommendation against (non-standardized formulations, low-quality evidence)
ESCEO	2019	Weak recommendation for short-term analgesia when patients have a contraindication for the use of NSAIDs or have insufficient analgesia on NSAID therapy	Weak recommendation for, only to be used when patients have a contraindication for the use of NSAIDs or have insufficient analgesia on NSAID therapy	Not included	Not included

AAOS—American Academy of Orthopedic Surgeons; ACR/AF—American College of Rheumatology/Arthritis Foundation; OARSI—Osteoarthritis Research Society International; ESCEO—European Society for Clinical and Economic Aspects of Osteoporosis, Osteoarthritis and Musculoskeletal Diseases; IACS—intra-articular corticosteroids; IAHA—intra-articular hyaluronic acid; PRP—platelet-rich plasma; MSCs—mesenchymal stem cells; NSAIDs—nonsteroidal anti-inflammatory drugs.

## References

[1] Hunter D.J., Bierma-Zeinstra S. (2019). Osteoarthritis. Lancet.

[2] Carlson A.K., Rawle R.A., Wallace C.W., Brooks E.G., Adams E., Greenwood M.C., Olmer M., Lotz M.K., Bothner B., June R.K. (2019). Characterization of Synovial Fluid Metabolomic Phenotypes of Cartilage Morphological Changes Associated with Osteoarthritis. Osteoarthr. Cartil..

[3] Nguyen U.-S.D.T., Zhang Y., Zhu Y., Niu J., Zhang B., Felson D.T. (2011). Increasing Prevalence of Knee Pain and Symptomatic Knee Osteoarthritis: Survey and Cohort Data. Ann. Intern. Med..

[4] Zhang Y., Jordan J.M. (2010). Epidemiology of Osteoarthritis. Clin. Geriatr. Med..

[5] GBD 2015 Disease and Injury Incidence and Prevalence Collaborators (2016). Global, Regional, and National Incidence, Prevalence, and Years Lived with Disability for 310 Diseases and Injuries, 1990–2015: A Systematic Analysis for the Global Burden of Disease Study 2015. Lancet.

[6] Bannuru R.R., Osani M.C., Vaysbrot E.E., Arden N.K., Bennell K., Bierma-Zeinstra S.M.A., Kraus V.B., Lohmander L.S., Abbott J.H., Bhandari M. (2019). OARSI Guidelines for the Non-Surgical Management of Knee, Hip, and Polyarticular Osteoarthritis. Osteoarthr. Cartil..

[7] Kolasinski S.L., Neogi T., Hochberg M.C., Oatis C., Guyatt G., Block J., Callahan L., Copenhaver C., Dodge C., Felson D. (2020). 2019 American College of Rheumatology/Arthritis Foundation Guideline for the Management of Osteoarthritis of the Hand, Hip, and Knee. Arthritis Care Res..

[8] Jevsevar D.S. (2013). Treatment of Osteoarthritis of the Knee: Evidence-Based Guideline, 2nd Edition. J. Am. Acad. Orthop. Surg..

[9] Bruyère O., Honvo G., Veronese N., Arden N.K., Branco J., Curtis E.M., Al-Daghri N.M., Herrero-Beaumont G., Martel-Pelletier J., Pelletier J.P. (2019). An Updated Algorithm Recommendation for the Management of Knee Osteoarthritis from the European Society for Clinical and Economic Aspects of Osteoporosis, Osteoarthritis and Musculoskeletal Diseases (ESCEO). Semin. Arthritis Rheum..

[10] Arden N.K., Perry T.A., Bannuru R.R., Bruyère O., Cooper C., Haugen I.K., Hochberg M.C., McAlindon T.E., Mobasheri A., Reginster J.Y. (2021). Non-Surgical Management of Knee Osteoarthritis: Comparison of ESCEO and OARSI 2019 Guidelines. Nat. Rev. Rheumatol..

[11] Highlights of Prescribing Information Acetaminophen Injection, for Intravenous Use. https://www.accessdata.fda.gov/drugsatfda_docs/label/2015/204767s000lbl.pdf.

[12] Conaghan P.G., Arden N., Avouac B., Migliore A., Rizzoli R. (2019). Safety of Paracetamol in Osteoarthritis: What Does the Literature Say?. Drugs Aging.

[13] Machado G.C., Maher C.G., Ferreira P.H., Pinheiro M.B., Lin C.-W.C., Day R.O., McLachlan A.J., Ferreira M.L. (2015). Efficacy and Safety of Paracetamol for Spinal Pain and Osteoarthritis: Systematic Review and Meta-Analysis of Randomised Placebo Controlled Trials. BMJ.

[14] Leopoldino A.O., Machado G.C., Ferreira P.H., Pinheiro M.B., Day R., McLachlan A.J., Hunter D.J., Ferreira M.L. (2019). Paracetamol versus Placebo for Knee and Hip Osteoarthritis. Cochrane Database Syst. Rev..

[15] Jung S.Y., Jang E.J., Nam S.W., Kwon H.H., Im S.G., Kim D., Cho S.K., Kim D., Sung Y.K. (2018). Comparative Effectiveness of Oral Pharmacologic Interventions for Knee Osteoarthritis: A Network Meta-Analysis. Mod. Rheumatol..

[16] FDA Drug Safety Communication: Prescription Acetaminophen Products to Be Limited to 325 Mg per Dosage Unit; Boxed Warning Will Highlight Potential for Severe Liver Failure|FDA. https://www.fda.gov/drugs/drug-safety-and-availability/fda-drug-safety-communication-prescription-acetaminophen-products-be-limited-325-mg-dosage-unit.

[17] Charlesworth J., Fitzpatrick J., Perera N.K.P., Orchard J. (2019). Osteoarthritis—A Systematic Review of Long-Term Safety Implications for Osteoarthritis of the Knee. BMC Musculoskelet. Disord..

[18] Bruyère O., Cooper C., Pelletier J.P., Maheu E., Rannou F., Branco J., Luisa Brandi M., Kanis J.A., Altman R.D., Hochberg M.C. (2016). A Consensus Statement on the European Society for Clinical and Economic Aspects of Osteoporosis and Osteoarthritis (ESCEO) Algorithm for the Management of Knee Osteoarthritis-From Evidence-Based Medicine to the Real-Life Setting. Semin. Arthritis Rheum..

[19] Roberts E., Nunes V.D., Buckner S., Latchem S., Constanti M., Miller P., Doherty M., Zhang W., Birrell F., Porcheret M. (2016). Paracetamol: Not as Safe as We Thought? A Systematic Literature Review of Observational Studies. Ann. Rheum. Dis..

[20] Freye E., Levy J.V. (2008). Mechanism of Action of Opioids and Clinical Effects. Opioids in Medicine.

[21] Welsch P., Sommer C., Schiltenwolf M., Häuser W. (2015). Opioide Bei Chronischen Nicht-Tumorbedingten Schmerzen—Sind Sie Nichtopioidanalgetika Überlegen?. Der Schmerz.

[22] Katz J.N., Smith S.R., Collins J.E., Solomon D.H., Jordan J.M., Hunter D.J., Suter L.G., Yelin E., Paltiel A.D., Losina E. (2016). Cost-Effectiveness of Nonsteroidal Anti-Inflammatory Drugs and Opioids in the Treatment of Knee Osteoarthritis in Older Patients with Multiple Comorbidities. Osteoarthr. Cartil..

[23] Toupin April K., Bisaillon J., Welch V., Maxwell L.J., Jüni P., Rutjes A.W.S., Husni M.E., Vincent J., El Hindi T., Wells G.A. (2019). Tramadol for Osteoarthritis. Cochrane Database Syst. Rev..

[24] Welsch P., Petzke F., Klose P., Häuser W. (2020). Opioids for Chronic Osteoarthritis Pain: An Updated Systematic Review and Meta-Analysis of Efficacy, Tolerability and Safety in Randomized Placebo-Controlled Studies of at Least 4 Weeks Double-Blind Duration. Eur. J. Pain.

[25] Osani M.C., Lohmander L.S., Bannuru R.R. (2020). Is There Any Role for Opioids in the Management of Knee and Hip Osteoarthritis? A Systematic Review and Meta-Analysis. Arthritis Care Res..

[26] Sostres C., Gargallo C.J., Arroyo M.T., Lanas A. (2010). Adverse Effects of Non-Steroidal Anti-Inflammatory Drugs (NSAIDs, Aspirin and Coxibs) on Upper Gastrointestinal Tract. Best Pract. Res. Clin. Gastroenterol..

[27] Gunter B.R., Butler K.A., Wallace R.L., Smith S.M., Harirforoosh S. (2017). Non-Steroidal Anti-Inflammatory Drug-Induced Cardiovascular Adverse Events: A Meta-Analysis. J. Clin. Pharm. Ther..

[28] Grosser T., Ricciotti E., FitzGerald G.A. (2017). The Cardiovascular Pharmacology of Nonsteroidal Anti-Inflammatory Drugs. Trends Pharmacol. Sci..

[29] Steinmeyer J., Bock F., Stöve J., Jerosch J., Flechtenmacher J. (2018). Pharmacological Treatment of Knee Osteoarthritis: Special Considerations of the New German Guideline. Orthop. Rev..

[30] Holt R.J., Fort J.G., Grahn A.Y., Kent J.D., Bello A.E. (2015). Onset and Durability of Pain Relief in Knee Osteoarthritis: Pooled Results from Two Placebo Trials of Naproxen/Esomeprazole Combination and Celecoxib. Phys. Sportsmed..

[31] Nissen S.E., Yeomans N.D., Solomon D.H., Lüscher T.F., Libby P., Husni M.E., Graham D.Y., Borer J.S., Wisniewski L.M., Wolski K.E. (2016). Cardiovascular Safety of Celecoxib, Naproxen, or Ibuprofen for Arthritis. N. Engl. J. Med..

[32] Gregori D., Giacovelli G., Minto C., Barbetta B., Gualtieri F., Azzolina D., Vaghi P., Rovati L.C. (2018). Association of Pharmacological Treatments with Long-Term Pain Control in Patients with Knee Osteoarthritis: A Systematic Review and Meta-Analysis. JAMA.

[33] da Costa B.R., Reichenbach S., Keller N., Nartey L., Wandel S., Jüni P., Trelle S. (2017). Effectiveness of Non-Steroidal Anti-Inflammatory Drugs for the Treatment of Pain in Knee and Hip Osteoarthritis: A Network Meta-Analysis. Lancet.

[34] Jevsevar D.S., Shores P.B., Mullen K., Schulte D.M., Brown G.A., Cummins D.S. (2018). Mixed Treatment Comparisons for Nonsurgical Treatment of Knee Osteoarthritis: A Network Meta-Analysis. J. Am. Acad. Orthop. Surg..

[35] Primorac D., Molnar V., Rod E., Jeleč Ž., Čukelj F., Matišić V., Vrdoljak T., Hudetz D., Hajsok H., Borić I. (2020). Knee Osteoarthritis: A Review of Pathogenesis and State-of-the-Art Non-Operative Therapeutic Considerations. Genes.

[36] Henrotin Y., Mathy M., Sanchez C., Lambert C. (2010). Chondroitin Sulfate in the Treatment of Osteoarthritis: From in Vitro Studies to Clinical Recommendations. Ther. Adv. Musculoskelet. Dis..

[37] McAlindon T.E.E., Bannuru R.R.R., Sullivan M.C.C., Arden N.K.K., Berenbaum F., Bierma-Zeinstra S.M.M., Hawker G.A.A., Henrotin Y., Hunter D.J.J., Kawaguchi H. (2014). OARSI Guidelines for the Non-Surgical Management of Knee Osteoarthritis. Osteoarthr. Cartil..

[38] Simental-Mendía M., Sánchez-García A., Vilchez-Cavazos F., Acosta-Olivo C.A., Peña-Martínez V.M., Simental-Mendía L.E. (2018). Effect of Glucosamine and Chondroitin Sulfate in Symptomatic Knee Osteoarthritis: A Systematic Review and Meta-Analysis of Randomized Placebo-Controlled Trials. Rheumatol. Int..

[39] Zhu X., Sang L., Wu D., Rong J., Jiang L. (2018). Effectiveness and Safety of Glucosamine and Chondroitin for the Treatment of Osteoarthritis: A Meta-Analysis of Randomized Controlled Trials. J. Orthop. Surg. Res..

[40] Beaudart C., Lengelé L., Leclercq V., Geerinck A., Sanchez-Rodriguez D., Bruyère O., Reginster J.Y. (2020). Symptomatic Efficacy of Pharmacological Treatments for Knee Osteoarthritis: A Systematic Review and a Network Meta-Analysis with a 6-Month Time Horizon. Drugs.

[41] Honvo G., Bruyère O., Geerinck A., Veronese N., Reginster J.-Y. (2019). Efficacy of Chondroitin Sulfate in Patients with Knee Osteoarthritis: A Comprehensive Meta-Analysis Exploring Inconsistencies in Randomized, Placebo-Controlled Trials. Adv. Ther..

[42] Bach-Rojecky L., Vadunec D., Žunić K., Kurija J., Šipicki S., Gregg R., Mikula I., Primorac D. (2019). Continuing War on Pain: A Personalized Approach to the Therapy with Nonsteroidal Anti-Inflammatory Drugs and Opioids. Per. Med..

[43] Vina E.R., Kwoh C.K. (2018). Epidemiology of Osteoarthritis. Curr. Opin. Rheumatol..

[44] Caudle K.E., Dunnenberger H.M., Freimuth R.R., Peterson J.F., Burlison J.D., Whirl-Carrillo M., Scott S.A., Rehm H.L., Williams M.S., Klein T.E. (2017). Standardizing Terms for Clinical Pharmacogenetic Test Results: Consensus Terms from the Clinical Pharmacogenetics Implementation Consortium (CPIC). Genet. Med..

[45] Dean E., Söderlund A. (2015). What Is the Role of Lifestyle Behaviour Change Associated with Non-Communicable Disease Risk in Managing Musculoskeletal Health Conditions with Special Reference to Chronic Pain?. BMC Musculoskelet. Disord..

[46] Saba R., Kaye A.D., Urman R.D. (2017). Pharmacogenomics in Pain Management. Anesthesiol. Clin..

[47] Ko T.M., Wong C.S., Wu J.Y., Chen Y.T. (2016). Pharmacogenomics for Personalized Pain Medicine. Acta Anaesthesiol. Taiwanica.

[48] Blanco G., Martínez C., Ladero J.M., Garcia-Martin E., Taxonera C., Gamito F.G., Diaz-Rubio M., Agundez J.A.G. (2008). Interaction of CYP2C8 and CYP2C9 Genotypes Modifies the Risk for Nonsteroidal Anti-Inflammatory Drugs-Related Acute Gastrointestinal Bleeding. Pharmacogenet. Genom..

[49] Bach-Rojecky L., Čutura T., Lozić M., Kliškinjić I.H., Matišić V., Primorac D. (2021). Personalized Anesthetic Pharmacology. Personalized Medicine in Anesthesia, Pain and Perioperative Medicine.

[50] Crews K.R., Monte A.A., Huddart R., Caudle K.E., Kharasch E.D., Gaedigk A., Dunnenberger H.M., Leeder J.S., Callaghan J.T., Samer C.F. (2021). Clinical Pharmacogenetics Implementation Consortium (CPIC) Guideline for CYP2D6, OPRM1, and COMT Genotype and Select Opioid Therapy. Clin. Pharmacol. Ther..

[51] Primorac D., Bach-Rojecky L., Vađunec D., Juginović A., Žunić K., Matišić V., Skelin A., Arsov B., Boban L., Erceg D. (2020). Pharmacogenomics at the Center of Precision Medicine: Challenges and Perspective in an Era of Big Data. Pharmacogenomics.

[52] Altman R.D., Barthel H.R. (2011). Topical Therapies for Osteoarthritis. Drugs.

[53] Derry S., Conaghan P., Da Silva J.A.P., Wiffen P.J., Moore R.A. (2016). Topical NSAIDs for Chronic Musculoskeletal Pain in Adults. Cochrane Database Syst. Rev..

[54] Concoff A., Rosen J., Fu F., Bhandari M., Boyer K., Karlsson J., Einhorn T.A., Schemitsch E. (2019). A Comparison of Treatment Effects for Nonsurgical Therapies and the Minimum Clinically Important Difference in Knee Osteoarthritis. JBJS Rev..

[55] Prince M.J., Wu F., Guo Y., Gutierrez Robledo L.M., O’Donnell M., Sullivan R., Yusuf S. (2015). The Burden of Disease in Older People and Implications for Health Policy and Practice. Lancet.

[56] Wongrakpanich S., Wongrakpanich A., Melhado K., Rangaswami J. (2018). A Comprehensive Review of Non-Steroidal Anti-Inflammatory Drug Use in the Elderly. Aging Dis..

[57] Rodriguez-Merchan E.C. (2018). Topical Therapies for Knee Osteoarthritis. Postgrad. Med..

[58] Ramamoorthy S., Cidlowski J.A. (2016). Corticosteroids. Rheum. Dis. Clin. N. Am..

[59] Jüni P., Hari R., Rutjes A.W.S., Fischer R., Silletta M.G., Reichenbach S., da Costa B.R. (2015). Intra-Articular Corticosteroid for Knee Osteoarthritis. Cochrane Database Syst. Rev..

[60] Deyle G.D., Allen C.S., Allison S.C., Gill N.W., Hando B.R., Petersen E.J., Dusenberry D.I., Rhon D.I. (2020). Physical Therapy versus Glucocorticoid Injection for Osteoarthritis of the Knee. N. Engl. J. Med..

[61] Henriksen M., Christensen R., Klokker L., Bartholdy C., Bandak E., Ellegaard K., Boesen M.P., Coumine Riis R.G., Bartels E.M., Bliddal H. (2015). Evaluation of the Benefit of Corticosteroid Injection before Exercise Therapy in Patients with Osteoarthritis of the Knee: A Randomized Clinical Trial. JAMA Intern. Med..

[62] O’Neill T.W., Parkes M.J., Maricar N., Marjanovic E.J., Hodgson R., Gait A.D., Cootes T.F., Hutchinson C.E., Felson D.T. (2016). Synovial Tissue Volume: A Treatment Target in Knee Osteoarthritis (OA). Ann. Rheum. Dis..

[63] McCabe P.S., Parkes M.J., Maricar N., Hutchinson C.E., Freemont A., O’Neill T.W., Felson D.T. (2017). Brief Report: Synovial Fluid White Blood Cell Count in Knee Osteoarthritis: Association With Structural Findings and Treatment Response. Arthritis Rheumatol..

[64] Saltychev M., Mattie R., McCormick Z., Laimi K. (2020). The Magnitude and Duration of the Effect of Intra-Articular Corticosteroid Injections on Pain Severity in Knee Osteoarthritis: A Systematic Review and Meta-Analysis. Am. J. Phys. Med. Rehabil..

[65] Phillips M., Vannabouathong C., Devji T., Patel R., Gomes Z., Patel A., Dixon M., Bhandari M. (2020). Differentiating Factors of Intra-Articular Injectables Have a Meaningful Impact on Knee Osteoarthritis Outcomes: A Network Meta-Analysis. Knee Surg. Sports Traumatol. Arthrosc..

[66] Wernecke C., Braun H.J., Dragoo J.L. (2015). The Effect of Intra-Articular Corticosteroids on Articular Cartilage. Orthop. J. Sports Med..

[67] Dragoo J.L., Danial C.M., Braun H.J., Pouliot M.A., Kim H.J. (2012). The Chondrotoxicity of Single-Dose Corticosteroids. Knee Surg. Sports Traumatol. Arthrosc..

[68] Altman R., Hackel J., Niazi F., Shaw P., Nicholls M. (2018). Efficacy and Safety of Repeated Courses of Hyaluronic Acid Injections for Knee Osteoarthritis: A Systematic Review. Semin. Arthritis Rheum..

[69] Altman R., Manjoo A., Fierlinger A., Niazi F., Nicholls M. (2015). The Mechanism of Action for Hyaluronic Acid Treatment in the Osteoarthritic Knee: A Systematic Review. BMC Musculoskelet. Disord..

[70] Miller L.E., Bhattacharyya S., Parrish W.R., Fredericson M., Bisson B., Altman R.D. (2019). Safety of Intra-Articular Hyaluronic Acid for Knee Osteoarthritis: Systematic Review and Meta-Analysis of Randomized Trials Involving More than 8000 Patients. Cartilage.

[71] Altman R., Bedi A., Manjoo A., Niazi F., Shaw P., Mease P. (2019). Anti-Inflammatory Effects of Intra-Articular Hyaluronic Acid: A Systematic Review. Cartilage.

[72] Bowman E.N., Hallock J.D., Throckmorton T.W., Azar F.M. (2018). Hyaluronic Acid Injections for Osteoarthritis of the Knee: Predictors of Successful Treatment. Int. Orthop..

[73] Miller L.E., Fredericson M., Altman R.D. (2020). Hyaluronic Acid Injections or Oral Nonsteroidal Anti-Inflammatory Drugs for Knee Osteoarthritis: Systematic Review and Meta-Analysis of Randomized Trials. Orthop. J. Sports Med..

[74] Nicholls M., Shaw P., Niazi F., Bhandari M., Bedi A. (2019). The Impact of Excluding Patients with End-Stage Knee Disease in Intra-Articular Hyaluronic Acid Trials: A Systematic Review and Meta-Analysis. Adv. Ther..

[75] Dohan Ehrenfest D.M., Andia I., Zumstein M.A., Zhang C.-Q., Pinto N.R., Bielecki T. (2019). Classification of Platelet Concentrates (Platelet-Rich Plasma-PRP, Platelet-Rich Fibrin-PRF) for Topical and Infiltrative Use in Orthopedic and Sports Medicine: Current Consensus, Clinical Implications and Perspectives. Muscle Ligaments Tendons J..

[76] Wu Q., Luo X., Xiong Y., Liu G., Wang J., Chen X., Mi B. (2020). Platelet-Rich Plasma versus Hyaluronic Acid in Knee Osteoarthritis: A Meta-Analysis with the Consistent Ratio of Injection. J. Orthop. Surg..

[77] Gato-Calvo L., Magalhaes J., Ruiz-Romero C., Blanco F.J., Burguera E.F. (2019). Platelet-Rich Plasma in Osteoarthritis Treatment: Review of Current Evidence. Ther. Adv. Chronic Dis..

[78] Trams E., Kulinski K., Kozar-Kaminska K., Pomianowski S., Kaminski R. (2020). The Clinical Use of Platelet-Rich Plasma in Knee Disorders and Surgery-A Systematic Review and Meta-Analysis. Life.

[79] Vilchez-Cavazos F., Millán-Alanís J.M., Blázquez-Saldaña J., Álvarez-Villalobos N., Peña-Martínez V.M., Acosta-Olivo C.A., Simental-Mendía M. (2019). Comparison of the Clinical Effectiveness of Single Versus Multiple Injections of Platelet-Rich Plasma in the Treatment of Knee Osteoarthritis: A Systematic Review and Meta-Analysis. Orthop. J. Sports Med..

[80] Migliorini F., Driessen A., Quack V., Sippel N., Cooper B., El Mansy Y., Tingart M., Eschweiler J. (2020). Comparison between Intra-Articular Infiltrations of Placebo, Steroids, Hyaluronic and PRP for Knee Osteoarthritis: A Bayesian Network Meta-Analysis. Arch. Orthop. Trauma Surg..

[81] Hohmann E., Tetsworth K., Glatt V. (2020). Is Platelet-Rich Plasma Effective for the Treatment of Knee Osteoarthritis? A Systematic Review and Meta-Analysis of Level 1 and 2 Randomized Controlled Trials. Eur. J. Orthop. Surg. Traumatol..

[82] Tang J.Z., Nie M.J., Zhao J.Z., Zhang G.C., Zhang Q., Wang B. (2020). Platelet-Rich Plasma versus Hyaluronic Acid in the Treatment of Knee Osteoarthritis: A Meta-Analysis. J. Orthop. Surg. Res..

[83] Han Y., Huang H., Pan J., Lin J., Zeng L., Liang G., Yang W., Liu J. (2019). Meta-Analysis Comparing Platelet-Rich Plasma vs Hyaluronic Acid Injection in Patients with Knee Osteoarthritis. Pain Med..

[84] Zhang H., Wang C., Li H., Huang Y., Li Z. (2018). Intra-Articular Platelet-Rich Plasma versus Hyaluronic Acid in the Treatment of Knee Osteoarthritis: A Meta-Analysis. Drug Des. Devel. Ther..

[85] Chen Z., Wang C., You D., Zhao S., Zhu Z., Xu M. (2020). Platelet-Rich Plasma versus Hyaluronic Acid in the Treatment of Knee Osteoarthritis: A Meta-Analysis. Medicine.

[86] Di Y., Han C., Zhao L., Ren Y. (2018). Is Local Platelet-Rich Plasma Injection Clinically Superior to Hyaluronic Acid for Treatment of Knee Osteoarthritis? A Systematic Review of Randomized Controlled Trials. Arthritis Res. Ther..

[87] Sun S.-F., Lin G.-C., Hsu C.-W., Lin H.-S., Liou I.-H.S., Wu S.-Y. (2021). Comparing Efficacy of Intraarticular Single Crosslinked Hyaluronan (HYAJOINT Plus) and Platelet-Rich Plasma (PRP) versus PRP Alone for Treating Knee Osteoarthritis. Sci. Rep..

[88] Zhao J., Huang H., Liang G., Zeng L., Yang W., Liu J. (2020). Effects and Safety of the Combination of Platelet-Rich Plasma (PRP) and Hyaluronic Acid (HA) in the Treatment of Knee Osteoarthritis: A Systematic Review and Meta-Analysis. BMC Musculoskelet. Disord..

[89] Karasavvidis T., Totlis T., Gilat R., Cole B.J. (2020). Platelet-Rich Plasma Combined with Hyaluronic Acid Improves Pain and Function Compared with Hyaluronic Acid Alone in Knee Osteoarthritis: A Systematic Review and Meta-Analysis. Arthroscopy.

[90] Belk J.W., Kraeutler M.J., Houck D.A., Goodrich J.A., Dragoo J.L., McCarty E.C. (2021). Platelet-Rich Plasma Versus Hyaluronic Acid for Knee Osteoarthritis: A Systematic Review and Meta-Analysis of Randomized Controlled Trials. Am. J. Sports Med..

[91] Wang A.T., Feng Y., Jia H.H., Zhao M., Yu H. (2019). Application of Mesenchymal Stem Cell Therapy for the Treatment of Osteoarthritis of the Knee: A Concise Review. World J. Stem Cells.

[92] Shariatzadeh M., Song J., Wilson S.L. (2019). The Efficacy of Different Sources of Mesenchymal Stem Cells for the Treatment of Knee Osteoarthritis. Cell Tissue Res..

[93] Peric V., Kottek T., Molnar V., Matišić V., Čukelj F., Primorac D. (2020). Mesenchymal Stem Cells In the Treatment Of Knee Osteoarthritis. J. Stem Cells Res. Dev. Ther..

[94] Ilas D.C., Churchman S.M., McGonagle D., Jones E. (2017). Targeting Subchondral Bone Mesenchymal Stem Cell Activities for Intrinsic Joint Repair in Osteoarthritis. Futur. Sci. OA.

[95] McGonagle D., Baboolal T.G., Jones E. (2017). Native Joint-Resident Mesenchymal Stem Cells for Cartilage Repair in Osteoarthritis. Nat. Rev. Rheumatol..

[96] Hudetz D., Borić I., Rod E., Jeleč Ž., Radić A., Vrdoljak T., Skelin A., Lauc G., Trbojević-Akmačić I., Plečko M. (2017). The Effect of Intra-Articular Injection of Autologous Microfragmented Fat Tissue on Proteoglycan Synthesis in Patients with Knee Osteoarthritis. Genes.

[97] Hudetz D., Borić I., Rod E., Jeleč Ž., Kunovac B., Polašek O., Vrdoljak T., Plečko M., Skelin A., Polančec D. (2019). Early Results of Intra-Articular Micro-Fragmented Lipoaspirate Treatment in Patients with Late Stages Knee Osteoarthritis: A Prospective Study. Croat. Med. J..

[98] Borić I., Hudetz D., Rod E., Jeleč Ž., Vrdoljak T., Skelin A., Polašek O., Plečko M., Trbojević-Akmačić I., Lauc G. (2019). A 24-Month Follow-Up Study of the Effect of Intra-Articular Injection of Autologous Microfragmented Fat Tissue on Proteoglycan Synthesis in Patients with Knee Osteoarthritis. Genes.

[99] Kim S.H., Ha C.W., Park Y.B., Nam E., Lee J.E., Lee H.J. (2019). Intra-Articular Injection of Mesenchymal Stem Cells for Clinical Outcomes and Cartilage Repair in Osteoarthritis of the Knee: A Meta-Analysis of Randomized Controlled Trials. Arch. Orthop. Trauma Surg..

[100] Kim S.H., Djaja Y.P., Park Y.B., Park J.G., Ko Y.B., Ha C.W. (2020). Intra-Articular Injection of Culture-Expanded Mesenchymal Stem Cells Without Adjuvant Surgery in Knee Osteoarthritis: A Systematic Review and Meta-Analysis. Am. J. Sports Med..

[101] Ma W., Liu C., Wang S., Xu H., Sun H., Fan X. (2020). Efficacy and Safety of Intra-Articular Injection of Mesenchymal Stem Cells in the Treatment of Knee Osteoarthritis: A Systematic Review and Meta-Analysis. Medicine.

[102] Song Y., Zhang J., Xu H., Lin Z., Chang H., Liu W., Kong L. (2020). Mesenchymal Stem Cells in Knee Osteoarthritis Treatment: A Systematic Review and Meta-Analysis. J. Orthop. Transl..

[103] Maheshwer B., Polce E.M., Paul K., Williams B.T., Wolfson T.S., Yanke A., Verma N.N., Cole B.J., Chahla J. (2021). Regenerative Potential of Mesenchymal Stem Cells for the Treatment of Knee Osteoarthritis and Chondral Defects: A Systematic Review and Meta-Analysis. Arthrosc. J. Arthrosc. Relat. Surg..

[104] Ha C.W., Park Y.B., Kim S.H., Lee H.J. (2019). Intra-Articular Mesenchymal Stem Cells in Osteoarthritis of the Knee: A Systematic Review of Clinical Outcomes and Evidence of Cartilage Repair. Arthrosc. J. Arthrosc. Relat. Surg..

[105] Di Matteo B., Vandenbulcke F., Vitale N.D., Iacono F., Ashmore K., Marcacci M., Kon E. (2019). Minimally Manipulated Mesenchymal Stem Cells for the Treatment of Knee Osteoarthritis: A Systematic Review of Clinical Evidence. Stem Cells Int..

[106] Polancec D., Zenic L., Hudetz D., Boric I., Jelec Z., Rod E., Vrdoljak T., Skelin A., Plecko M., Turkalj M. (2019). Immunophenotyping of a Stromal Vascular Fraction from Microfragmented Lipoaspirate Used in Osteoarthritis Cartilage Treatment and Its Lipoaspirate Counterpart. Genes.

[107] Sharma L. (2021). Osteoarthritis of the Knee. N. Engl. J. Med..

